# Immune Biomarker Response Depends on Choice of Experimental Pain Stimulus in Healthy Adults: A Preliminary Study

**DOI:** 10.1155/2012/538739

**Published:** 2012-11-20

**Authors:** Yenisel Cruz-Almeida, Christopher D. King, Shannon M. Wallet, Joseph L. Riley

**Affiliations:** ^1^Pain Research and Intervention Center of Excellence, University of Florida, P.O. Box 103628, Gainesville, FL 32610-3628, USA; ^2^Department of Community Dentistry and Behavioral Science, College of Dentistry, University of Florida, P.O. Box 103628, Gainesville, FL 32610-3628, USA; ^3^Department of Periodontology, College of Dentistry, University of Florida, P.O. Box 103628, Gainesville, FL 32610-3628, USA; ^4^Department of Oral Biology, College of Dentistry, University of Florida, P.O. Box 103628, Gainesville, FL 32610-3628, USA

## Abstract

Few studies in healthy subjects have examined the neuroimmune responses associated with specific experimental pain stimuli, while none has measured multiple biomarkers simultaneously. The aim of the present study was to compare the neuro-immune responses following two common experimental pain stimuli: cold pressor test (CPT) and focal heat pain (FHP). Eight adults participated in two counterbalanced experimental sessions of FHP or CPT with continuous pain ratings and blood sampling before and 30 minutes after the sessions. Despite similar pain intensity ratings (FHP = 42.2 ± 15.3; CPT = 44.5 ± 34.1; *P* = 0.871), CPT and FHP induced different neuro-immune biomarker responses. CPT was accompanied by significant increases in cortisol (*P* = 0.046) and anti-inflammatory cytokine IL-10 (*P* = 0.043) with significant decreases in several pro-inflammatory mediators (IL-1**β** (*P* = 0.028), IL-12 (*P* = 0.012), TNF-**α** (*P* = 0.039), and MCP-1 (*P* = 0.038)). There were nonsignificant biomarker changes during the FHP session. There were close to significant differences between the sessions for IL-1**β** (*P* = 0.081), IFN-**γ** (*P* = 0.072), and IL-12 (*P* = 0.053) with biomarkers decreasing after CPT and increasing after FHP. There were stronger associations between catastrophizing and most biomarkers after CPT compared to FHP. Our results suggest that CPT is a stressful and painful stimulus, while FHP is mostly a painful stimulus. Thus, each experimental pain stimulus can activate different neuro-immune cascades, which are likely relevant for the interpretation of studies in chronic pain conditions.

## 1. Introduction

The cross-talk between the nervous and immune systems is of special interest to pain researchers since immune-derived signaling molecules have been implicated in altered nociception [1] and chronic pain conditions [2] including fibromyalgia [3], osteoarthritis [4], migraine [5], and temporomandibular disorders [6]. The field of pain research relies on the administration of standardized noxious stimulation with response comparisons to translate animal studies to human mechanistic studies, and further translate findings from healthy subjects to patients with chronic pain [7–9]. However, measures of experimental pain have been shown to correlate only moderately across stimulus modalities [1, 10–13]. Also, human imaging studies show a differential pattern of brain activation between two of the most common experimental pain stimuli: the cold pressor test (CPT) and focal heat pain (FHP) stimulation [14]. CPT is mainly mediated by venous nociceptors [15] and cutaneous nociceptors [16], while FHP activates cutaneous nociceptors with varying threshold temperatures [17]. Indeed, the overlap of heritability between the CPT and FHP stimulation has been shown to be relatively small, supporting the idea that these experimental manipulations are likely distinct phenomena [18].

Additionally, the experimental pain model of “pain-inhibition-by-pain” or “conditioned pain modulation” (CPM) paradigm is frequently used to evaluate the endogenous pain modulatory capacity of the nervous system in healthy and diseased conditions. The two most commonly used conditioning stimuli are CPT and FHP [19], and the underlying physiological mechanisms activated in CPM likely depend on the choice of stimuli, further impacting study results, and interpretations. Thus, an important first step to adequately interpret findings using CPM is the characterization of the neuro-immune biomarker responses induced by these commonly used noxious stimuli.

Only two studies to date have examined more than one neuro-immune biomarker responses in relation to various experimental pain stimuli in healthy volunteers. Edwards and colleagues [20] found a significant *increase* in interleukin-6 (IL-6) concentrations after administration of a series of acute experimental pain stimulations. Also, Goodin and colleagues [21] recently reported significant increases in cortisol and *decreases* in the concentrations of the soluble tumor necrosis factor-*α* receptor II (sTNF*α*RII) responses following separate sessions of CPT and a hot water immersion task. The different directions of change of the neuro-immune biomarkers in these previous studies (i.e., *increase* in IL-6 [20] versus an increase in cortisol, but a *decrease* in sTNF*α*RII [21]) highlight the need for studies characterizing the immune responses induced by commonly employed experimental pain methodology in healthy adults. A limitation in these previous studies was the measurement of one biomarker at a time, which does not provide the spectrum of neuro-immune responses and could account for the conflicting results. Simultaneous measurement of multiple neuro-inflammatory mediators is crucial in understanding patterns of physiological processes activated by each experimental stimulus, especially since the neuro-immune mediators normally function as part of a complex physiological network with feedback mechanisms and overlapping biological roles [22, 23].

To date, no study has measured multiple neuro-inflammatory biomarkers simultaneously in response to specific experimental pain stimuli in healthy human subjects. Therefore, the present preliminary study examines for the first time a range of neuro-immune biomarkers in healthy human subjects responding to two different noxious stimuli. Our primary aim was to compare the immune responses between noxious focal heat (FHP) and cold pressor (CPT) pain stimulation on separate occasions. These are two of the most universally used stimuli in experimental pain research, especially in CPM paradigms. Results from the present investigation will advance our current understanding of the biomarkers and subsequent pathways activated by our experimental stimuli which are likely relevant for the interpretation of future studies in chronic pain conditions.

## 2. Materials and Methods

### 2.1. Participants

Four males and four females between the ages of 24 and 59 (mean = 37.9 ± 13.6) participated in a psychophysical study examining physiological responses to noxious heat and noxious cold. Exposure to thermal stimuli was randomized, counter balanced, and conducted on separate days. The University of Florida Institutional Review Board approved the research study. All individuals provided informed consent and were compensated for their participation.

### 2.2. Testing Sessions

Study participants arrived at the laboratory for testing between 1100 and 1300 hours to avoid the circadian rhythm variations associated with the neuro-endocrine biomarkers. Following a-10-minute rest period, baseline blood samples were collected by venipuncture. Following an additional 10 minutes of rest, three blood pressure measurements were taken using an Omron 780 Automatic Blood Pressure Monitor (Omron Healthcare), after which sensory testing began. Each participant underwent five 30-second (s) trials with 30-s rest periods in between. Pain was continuously rated during the trials using an electronic visual analogue scale (eVAS) with anchor points of 0 (no pain) and 100 (intolerable pain). Thirty minutes following the sensory testing trials, a second blood sample was collected, and a catastrophizing questionnaire was administered.

### 2.3. Focal Heat Pain (FHP) Stimulus

Focal thermal stimuli were administered to the left thenar eminence by an electronically held 23 mm × 23 mm Peltier-based thermode. Thermode temperature for the first trial was set to 48.0°C (males) or 47.0°C (females) based on previous studies tailoring individualized temperatures by gender [24]. If subjects rated the pain less than 30 out of 100 (eVAS), the temperature was adjusted by 1.0°C each trial until the subject rated the pain approximately 30 out of 100 on the eVAS. The target eVAS rating was between 30 and 60 for all subjects.

### 2.4. Cold Pressor Test (CPT) Stimulus

Subjects immersed their right foot in a water bath at 8.0°C (males) or 10.0°C (females) based on previous studies which tailored individual temperatures by gender [24]. If subjects rated the pain less than 30 out of 100 (eVAS), then the temperature was decreased by 2.0°C in the subsequent trial.

### 2.5. Catastrophizing Questionnaire

After each of the experimental sessions, an adaptation of the Pain Catastrophizing Scale (PCS) was administered to each participant. The PCS [25, 26] is a brief measure of catastrophizing related to pain. The instrument was used as revised by Edwards and colleagues [20] to assess situation-specific catastrophizing. The items were rated on a 5-point scale with anchors of 0: not at all and 4: all the time. The PCS has shown good internal consistency (Cronbach's *α* = 0.95) and test-retest reliability *r* = 0.75 [25]. 

### 2.6. Biomarker Detection

Plasma levels of soluble mediators (cortisol, *α*-MSH, IFN-*γ*, IL-1*β*, IL-6, IL-8, IL-10, IL-12(p70), MCP-1, and TNF-*α*) were qualitatively and quantitatively evaluated by multiplex assays (Millipore) according to the manufacturer's instructions. Data were acquired using a Luminex 200 and analyzed using a standard curve, 5 parameter logistics, and Milliplex software (Viagene). Data are expressed as changes in expression calculated by subtracting pretesting concentration from posttesting concentration, thus, positive values indicate an increase in concentrations, while negative values reflect a decrease in concentrations as a result of the pain modality. 

### 2.7. Statistical Analysis

Repeated measures analysis of variance (ANOVA) was performed to examine the relationship between testing modalities, pain ratings, and blood pressure measurements before, during, and after each experimental session. The nonparametric statistical analysis of Wilcoxon signed-rank test was performed on the change in score between modalities. Probability values between 0.001–0.05 were considered “statistically significant”; probability values between 0.051–0.099 were considered a “trend towards statistical significance”; probability values greater than 0.100 were considered non-statistically significant changes. Cohen's *d* effect sizes are presented where appropriate following the conventions of Cohen [27] for tests of adjusted mean differences.

## 3. Results

### 3.1. No Significant Differences in Peak Pain and Blood Pressure following CPT and FHP

Individualized temperatures for the focal heat pain (FHP) and cold pressor test (CPT) produced comparable levels of moderate pain, as determined by peak pain ratings (PPR) which were defined as the maximum pain ratings during the 30-second trial (mean FHP = 42.2 ± 15.3, mean CPT = 44.5 ± 34.1, and *P* = 0.871). A repeated measures ANOVA revealed no main effects of test trials within a session (*F* = 1.04, *P* = 0.436) or between sessions (CPT/FHP) (*F* = 0.03, *P* = 0.871). The interaction was also not statistically significant (*F* = 1.23, *P* = 0.357).

Changes in systolic blood pressure from baseline compared to values obtained after each 30-second trial revealed no main effects of trial (*F* = 1.39, *P* = 0.315), session (*F* = 0.41, *P* = 0.535), or their interaction (*F* = 0.21, *P* = 0.950) (estimated mean across trials for FHP = 115.5 ± 5.0, for CPT = 123.3 ± 5.2). Similarly, there were no significant changes in diastolic blood pressure depending on trial (*F* = 2.14, *P* = 0.152), session (*F* = 0.22, *P* = 0.649), or their interaction (0.70, *P* = 0.635) (estimated mean across trials for FHP = 66.9 ± 3.8; for CPT = 75.8. ± 3.6).

### 3.2. Alteration in Soluble Neuro-Immune Biomarkers following Cold Pressor Test (ΔCPT) but not Focal Heat Pain (ΔFHP)

In order to determine if pain can induce changes in immuno-modulatory soluble mediators, changes in cyto/chemokine plasma concentrations along with the hormones cortisol and *α*-MSH prior to and 30 minutes following FHP or CPT stimuli were determined after which the change in expression was calculated (Table 1). While cortisol concentrations significantly increased after the CPT session (*P* = 0.046), there was no change in the concentration of the neuropeptide *α*-MSH (*P* = 0.841). On the other hand, while concentrations of the cytokine IL-10 significantly increased following the CPT session (*P* = 0.043), the concentrations of the cytokines IL-1*β*, TNF-*α*, MCP-1, and IL-12p70 significantly decreased after the CPT session (*P* < 0.05). Although not statistically significant, there was also a trend towards a decrease in the levels of IFN-*γ* and IL-8 (*P* < 0.099), while there was no change in the concentration of IL-6 after the CPT session (*P* > 0.100). Unlike the CPT session, no changes in the concentration of any soluble mediators evaluated were observed following the FHP session, although there was an increase in cortisol concentrations that was also not statistically significant (*P* = 0.128).

### 3.3. A More Robust Neuro-Immune Response to CPT Compared to FHP

To address the primary aim of our study, we compared the changes in cortisol and biomarker concentrations between the experimental CPT and FHP sessions (ΔCPT/ΔFHP) (Table 1). Cortisol and immune biomarkers baseline concentrations were compared between the experimental CPT and FHP sessions where mean baseline values for all biomarkers measured were not found to be statistically different across the two experimental modalities (*P* > 0.05). There was a trend towards statistical significant differences in cortisol concentrations between the ΔCPT and ΔFHP sessions (*P* = 0.066) with greater cortisol elevations following CPT than after FHP. Similarly, there was a trend toward statistical significance in concentration differences between the CPT and FHP sessions of IL-1*β* (*P* = 0.081), IFN-*γ* (*P* = 0.072), and IL-12 (*P* = 0.053) cytokine concentrations with cytokine concentrations decreasing after CPT and increasing after FHP.

### 3.4. Changes in Cortisol Were Related to Pain Ratings, Catastrophizing Scores and Immune Biomarker Changes

Although statistical significance was not reached, changes in cortisol concentrations were modestly correlated with the average peak pain ratings within the CPT (*r* = 0.46, *P* = 0.251) and FHP (*r* = 0.40, *P* = 0.326) sessions. Changes in cortisol concentrations were also significantly correlated to the PCS scores within the CPT session (*r* = 0.75, *P* = 0.023), while within the FHP session the correlation between the PCS scores and the cortisol changes was modest in magnitude and did not reach statistical significance (*r* = 0.44, *P* = 0.275). Similarly, immune biomarker concentration changes were also moderately correlated to the changes in cortisol concentrations and average peak pain ratings (summarized in Table 2).

## 4. Discussion

We sought to compare the neuro-immune responses associated with two commonly used experimental pain stimuli: CPT and FHP. Our findings suggest that despite similar pain intensity ratings, CPT and FHP stimulation induced different neuro-immune responses. Significant decreases in the pro-inflammatory mediators IL-1*β*, IFN-*γ*, IL-12p70, IL-8, TNF-*α*, and MCP-1 in combination with significant increases in the cytokine IL-10 after CPT are likely a result of the activation hypothalamic-pituitary-adrenal (HPA) axis (i.e., stress response). This is supported by the modest to strong correlation coefficients between cortisol and the different immune biomarkers. The significant increase in cortisol produced by CPT appears to be analogous to an acute high stress condition where high systemic glucocorticoid concentrations saturate glucocorticoid receptors and exert suppressive effects on the immune response to prevent autoimmune damage [28, 29]. The saturation of glucocorticoid receptors directly downregulate the transcription of IL-1*β* and TNF-*α* genes and indirectly upregulate the transcription of the NF-*κ*B inhibitor, I*κβ*, further decreasing transcription of several pro-inflammatory cytokines [28, 30].

In contrast, FHP only modestly induced HPA axis activation with nonsignificant cortisol elevations. This appears similar to basal or low stress conditions where low concentrations of systemic glucocorticoids only partially activate the glucocorticoid receptor leading to early increases in the immune response or a permissive effect [28, 30]. Also, of note was that biomarker concentration changes for FHP and CPT were generally in opposite directions. In general, there were stronger correlations between most neuro-immune biomarkers and the levels of catastrophizing after the CPT session compared to the FHP session. Therefore, our results may suggest that CPT is a stronger stressor than FHP as administered in most pain studies, despite equivalent levels of pain.

Consistent with our findings in healthy subjects, Edwards and colleagues [20] reported significant increases in cortisol and IL-6 after a series of experimental painful stimulations that included CPT and FHP. Pain catastrophizing was also significantly associated with IL-6, but not cortisol reactivity over a period of one hour. However, both stimuli were given simultaneously during one session; thus, the stimulus-specific responses could not be discerned. Goodin and colleagues [21] also reported a significantly stronger cortisol elevation after the CPT compared to other stimuli including a hot water task. In the present study, there were also non-significant increases in the cytokine IL-6 following CPT and FHP with a significant decrease in the pro-inflammatory cytokine TNF-*α* only after the CPT session. Most pain studies including those in clinical pain samples present IL-6 only as a pro-inflammatory cytokine, but IL-6 is a pleiotropic cytokine that can inhibit IL-1 and TNF-*α*, thus, having opposing pro- and anti-inflammatory profiles [28, 31]. Other studies using nonpainful stressors have reported that stress-induced glucocorticoid activation suppresses IL-1*β* and TNF-*α* production, but not IL-6. In general, IL-6 seems to be more resistant to glucocorticoid signaling [32–38]; therefore, in studies where a limited number of immune biomarkers can be measured, other cytokines in addition to IL-6 may be more informative of the pro-inflammatory response.

Thus, the assumption that pain is always a pro-inflammatory stimulus is not consistent to the findings in the stress literature. Our study specifically supports the idea that CPT is a painful stimulus and a strong stressor which downregulates the acute immune response, while FHP is also a painful stimulus, but less of a stressor with permissive immune responses. Two findings are consistent with this assertion: (1) pain ratings did not differ between CPT and FHP and (2) catastrophizing was more related to the changes in biomarkers within the CPT session compared to the FHP. This is directly relevant to the interpretation of pain-inhibition-by-pain paradigms, which may use either CPT or FHP as the conditioning stimulus. Whether stress-induced analgesia is needed for the CPM paradigm is not currently known. Future studies should compare various conditioning stimuli in separate sessions along with the measurement of multiple neuro-immune biomarkers to elucidate the putative CPM mechanisms in healthy participants and their relationship to stimulus choice. In addition, our findings may be related to the size of the area under stimulation with CPT involving a greater overall skin area, thus, strongly activating the stress system. Since the skin can mount a local stress response including HPA axis mediators such as CRH in response to stressful stimuli [39, 40], greater skin area stimulated may translate into a greater stress responses. Future studies should use testing methods which standardize the area of skin contact.

The present study reflects biomarker changes that occurred 30 minutes postexperimental pain testing in a relatively small number of participants. Significant increases in cortisol can be measured within 30 minutes of experimental pain application, while at least an hour is needed to measure significant increases in IL-6 after painful stimuli administration [20]. Changes in cytokines have been reported to be greater 30 to 120 minutes after application of a nonpainful stressor compared to immediately after the application of a nonpainful stressor [41]. The time course of cytokine responses following experimental pain procedures is not currently known, thus, future studies should include measurements at additional time points in a larger sample of healthy subjects. In addition, to characterize the changes in the pain system, other biomarkers should be measured including sensory neuropeptides (i.e., nerve growth factor (NGF)) and mediators along the NF-*κ*B pathway, which have been implicated in chronic inflammatory pain conditions such as arthritis [42, 43].

## 5. Conclusions

The present investigation provides a preliminary framework measuring the relationship between experimental pain stimulation and neuro-immune responses using a systems-based approach in healthy subjects. The main advantage of the present investigation was the measurement of multiple biomarkers simultaneously, rather than a few biomarkers selected on statistical significance. Our results suggest that CPT and FHP experimental models set in motion different physiological responses with CPT engaging the stress and pain systems and FHP engaging mostly the pain system. Therefore, our results emphasize the importance of knowing what mechanisms are activated or inhibited by each experimental pain paradigm. Such information will aid in the determination of which experimental modality might be the most useful to study specific chronic pain conditions.

## Figures and Tables

**Table 1:** Biomarker concentrations changes (post-pre) after cold pain (ΔCPT) and after hot pain (ΔFHP) stimulation.

** **	ΔCPT* (pg/mL)	ΔCPT *P* ^*¥*^	ΔFHP* (pg/mL)	ΔFHP *P* ^*¥*^	ΔCPT/ΔFHP
	(Mean ± SD)		(Mean ± SD)		*P* ^*¥*^, *d* ^*£*^

Cortisol	**81,127 ± 123,994**	**0.046**	38,741 ± 87,179	0.128	**0.066, 0.3**
IL-1*β*	**−2.20 ± 3.6**	**0.028**	−0.04 ± 1.0	0.398	**0.081, 0.8**
TNF-*α*	**−0.50 ± 0.7**	**0.039**	0.06 ± 0.6	0.889	0.236, 0.8
IFN-*γ*	**−2.89 ± 4.5**	**0.091**	6.04 ± 15.7	0.310	**0.072, 0.7**
IL-8	**−0.84 ± 0.7**	**0.075**	5.33 ± 10.1	0.889	0.345, 0.8
IL-12	**−4.57 ± 10.1**	**0.012**	14.27 ± 36.9	0.310	**0.053, 0.6**
MCP-1	**−31.29 ± 43.2**	**0.038**	−3.80 ± 47.2	0.161	0.606, 0.6
*α*MSH	6.25 ± 57.1	0.841	−13.90 ± 5.6	0.176	0.476, 0.4
IL-6	1.80 ± 5.7	0.398	2.95 ± 10.3	0.727	0.674, 0.1
IL-10	**29.00 ± 102.6**	**0.043**	−0.11 ± 6.3	0.962	0.426, 0.4

*Positive values indicate an increase in biomarker concentrations, while negative values represent a decrease in biomarker concentrations.

^¥^Statistical probability.

^*£*^Cohen's effect sizes are generally defined as small (*d* = 0.2), medium (*d* = 0.5), and large (*d* = 0.8) effects.

**Table 2:** Bivariate correlations between cortisol, catastrophizing and pain ratings with immune biomarker concentrations changes (post-pre) after cold pain (ΔCPT) and hot pain (ΔFHP) stimulation.

	Cortisol	Cortisol	PCS^€^	PCS	PPR^*£*^	PPR
	ΔCPT*	ΔFHP*	ΔCPT	ΔFHP	ΔCPT	ΔFHP
	*r* (*P*-value)	*r* (*P*-value)	*r* (*P*-value)	*r* (*P*-value)	*r* (*P*-value)	*r* (*P*-value)

IL-1*β*	−0.57 (0.140)	0.31 (0.455)	−0.64 (0.087)	0.22 (0.601)	−0.35 (0.395)	0.64 (0.087)
TNF-*α*	−0.38 (0.353)	0.20 (0.635)	−0.29 (0.486)	0.18 (0.670)	−0.46 (0.251)	0.26 (0.534)
IFN-*γ*	−0.34 (0.410)	0.24 (0.567)	−0.18 (0.670)	0.15 (0.723)	−0.25 (0.550)	0.63 (0.094)
IL-8	−0.70 (0.053)	0.17 (0.687)	−0.52 (0.187)	0.22 (0.601)	−0.50 (0.207)	0.64 (0.087)
IL-12	−0.46 (0.251)	0.25 (0.550)	−0.35 (0.395)	0.16 (0.705)	−0.41 (0.313)	0.65 (0.081)
MCP-1	−0.58 (0.132)	0.02 (0.963)	−0.36 (0.381)	0.33 (0.425)	−0.49 (0.218)	0.85 (0.008)
*α*MSH	0.05 (0.906)	0.20 (0.635)	0.08 (0.851)	0.00 (0.998)	0.17 (0.687)	0.13 (0.759)
IL-6	0.64 (0.087)	0.50 (0.207)	0.44 (0.275)	0.16 (0.705)	0.55 (0.158)	0.65 (0.081)
IL-10	0.30 (0.470)	−0.36 (0.381)	0.22 (0.601)	−0.49 (0.218)	0.35 (0.395)	−0.64 (0.087)

*Positive values indicate an increase in biomarker concentrations, while negative values represent a decrease in biomarker concentrations.

^€^PCS = Pain Catastrophizing Scale scores.

^*£*^PPR = Peak Pain Ratings.
